# Post-COVID Condition and Neuroinflammation: Possible Management with Antioxidants

**DOI:** 10.3390/antiox14070840

**Published:** 2025-07-08

**Authors:** Noemí Cárdenas-Rodríguez, Iván Ignacio-Mejía, César Miguel Mejía-Barradas, Daniel Ortega-Cuellar, Felipe Muñoz-González, Marco Antonio Vargas-Hernández, Exsal Manuel Albores-Méndez, Gabriela Ibáñez-Cervantes, Roberto Medina-Santillán, Aarón Hernández-Ortiz, Elizabeth Herrera-López, Cindy Bandala

**Affiliations:** 1 Laboratorio de Neurociencias, Instituto Nacional de Pediatriía, Secretariía de Salud, Mexico City 04530, Mexico; ncardenasr@pediatria.gob.mx; 2 Sección de Investigación, Escuela Militar de Graduados de Sanidad, Centro de Investigación y Desarrollo del Ejército y Fuerza Aérea Mexicanos, Universidad del Ejército y Fuerza Aérea, Mexico City 11200, Mexico; ivanignacio402@gmail.com (I.I.-M.); mavh78@yahoo.com.mx (M.A.V.-H.); albores_09@hotmail.com (E.M.A.-M.); 3 Escuela Superior de Medicina, Instituto Politécnico Nacional, Mexico City 11340, Mexico; cmejiab@ipn.mx (C.M.M.-B.); xilipkoru@gmail.com (F.M.-G.); gibanezc@ipn.mx (G.I.-C.); robertomedsan@yahoo.com (R.M.-S.); aahernandezo14@gmail.com (A.H.-O.); 4 Laboratorio de Nutrición Experimental, Instituto Nacional de Pediatría, Secretaría de Salud, Mexico City 04530, Mexico; dortegadan@gmail.com; 5 Departamento de Investigación, Universidad Estatal del Valle de Ecatepec, Mexico City 55210, Mexico; eliza_herrera83@yahoo.com.mx

**Keywords:** post-COVID condition, neuroinflammation, antioxidants

## Abstract

Post-COVID condition (PCC) is a complex syndrome characterized by the persistence of diverse symptoms—including respiratory, neurological, and psychiatric manifestations—that last for weeks or months after acute Severe Acute Respiratory Syndrome Coronavirus 2 (SARS-CoV-2) infection. Epidemiological data indicate a higher prevalence among women and older adults, with significant impacts on daily functioning. The pathophysiology of PCC is multifactorial, involving immune dysregulation, viral persistence, mitochondrial dysfunction, and oxidative stress, all of which contribute to sustained neuroinflammation. This narrative review examines the clinical features, risk factors, and current evidence on antioxidant-based interventions as potential therapeutic strategies for PCC. A wide range of compounds—including vitamins, polyphenols, and endogenous antioxidants—have shown promise in mitigating neuroinflammation and oxidative damage in both clinical and experimental settings. Antioxidants may help restore redox balance and improve neurological outcomes in affected patients. However, further clinical research is essential to determine their efficacy, safety, and optimal therapeutic protocols.

## 1. Introduction

Severe Acute Respiratory Syndrome Coronavirus 2 (SARS-CoV-2) infection induces a broad spectrum of systemic and neurological symptoms. Following the acute phase of the disease, approximately 87% of patients continue to experience symptoms, a condition known as post-COVID-19 condition (PCC), or long COVID [1].

Several definitions have been proposed for PCC, generally based on the persistence or new onset of symptoms after the acute phase of COVID-19 and the exclusion of alternative diagnoses. Correa and Vallespin define PCC as the presence of symptoms in individuals with a history of confirmed SARS-CoV-2 infection, which persist or newly appear approximately three months after the initial illness, last for at least two months, and cannot be explained by another diagnosis [2].

Among the most widely accepted definitions, the World Health Organization (WHO) defines PCC as the continuation or onset of new symptoms three months after infection, lasting at least two months and not attributable to other causes [3]. Similarly, the National Institute for Health and Care Excellence (NICE) defines it as symptoms lasting more than 12 weeks that cannot be explained by other conditions [4]. The Centers for Disease Control and Prevention (CDC) offers a broader description, referring to persistent or newly emerging signs, symptoms, and complications following acute COVID-19 [5].

The most common symptoms of PCC include chest pain, fatigue, dyspnea, cough, cognitive impairment, and other related psychiatric conditions, including depression and anxiety [6,7]. The mechanisms underlying symptom persistence in PCC remain unclear; however, one hypothesis suggests that prolonged inflammation due to SARS-CoV-2 persistence in the body contributes to this condition [8,9,10]. Systemic inflammation, neuroinflammation, microvascular injury, and thrombosis are critical factors in PCC development [11,12]. Among these, the NOD-like receptor family pyrin domain-containing 3 (NLRP3) inflammasome plays a prominent role, leading to a cytokine storm characterized by elevated levels of interleukin (IL)-1β, interleukin-2 (IL-2), interleukin-6 (IL-6), interleukin-17 (IL-17), tumor necrosis factor (TNF)-α, interferon (IFN)-γ, C-X-C motif chemokine ligand 10 (CXCL10), and C-C motif chemokine ligands 2 (CCL2) and 3 (CCL3) [12,13].

This dysregulated immune response also results in oxidative stress with the activation of NADPH oxidase (NOX) and an increase in reactive oxygen species (ROS), along with reduced antioxidant defenses and increased mitochondrial damage [14]. Persistent hyperinflammation and oxidative stress, induced by COVID-19 antigens, are key contributors to the pathophysiology of PCC [15]. Many PCC symptoms may be centrally mediated by the brain such as fatigue, malaise, fever, dyspnea, cognitive, and neurological impairments, and most notably, “brain fog”, defined as a subjective sense of mental confusion, cognitive clouding, or difficulty concentrating. It may manifest with symptoms including frequent forgetfulness, mental sluggishness, trouble articulating ideas, or feeling that simple tasks require excessive effort [16,17].

Therefore, this review evaluates current scientific evidence regarding the role of antioxidant compounds as potential adjuvant therapies for neurological and psychiatric symptoms associated with neuroinflammation in PCC. Adjuvant therapy refers to any therapeutic intervention administered in conjunction with standard care, intended to enhance treatment efficacy, modulate pathological mechanisms, such as inflammation and oxidative stress, or alleviate persistent symptoms [18,19,20]. Given that there is no universally established standard of care at present for neuropsychiatric manifestations in PCC, adjuvant strategies—particularly those involving natural antioxidant and anti-inflammatory compounds—are of increasing interest in mitigating long-term sequelae [19,20].

A comprehensive literature search was conducted for this review across major scientific databases, including PubMed, ProQuest, EBSCO, Scopus, ScienceDirect, Web of Science, PubChem, NCBI Bookshelf, DrugBank, and ClinicalTrials.gov. The search strategy aimed to identify peer-reviewed studies and authoritative sources relevant to PCC, neuroinflammation, and antioxidant interventions. A broad range of publication types was considered, including original research articles, narrative and systematic reviews, meta-analyses, clinical trials, books, and entries from specialized databases. The search terms included combinations of keywords such as “COVID-19”, “post-COVID condition”, “long COVID”, “neuroinflammation”, “oxidative stress”, “reactive oxygen species”, “free radicals”, “mitochondrial dysfunction”, “antioxidant compounds”, and “antioxidant enzymes”. Study selection was performed iteratively, based on thematic relevance, scientific rigor, and each study’s contribution to the manuscript’s overall objective. A total of 199 references were included, reflecting the breadth, diversity, and current state of knowledge in this field.

## 2. Clinical Presentation of PCC

PCC, or long COVID, manifests through a heterogeneous set of symptoms that affect multiple organ systems and persist beyond the acute phase of SARS-CoV-2 infection. These symptoms have been documented in patients with varying degrees of initial illness severity, from those hospitalized with severe disease to individuals with mild or even asymptomatic cases [21]. Epidemiological analyses estimate a global prevalence of PCC of approximately 43%, with higher incidence reported in hospitalized individuals (54%) compared to non-hospitalized patients (34%) [22]. Women are more commonly affected than men, and older adults, particularly those above 40 years, and show increased susceptibility [23,24,25,26]. Risk factors also include pre-existing conditions such as asthma and allergic rhinitis [27], smoking status [28,29], and hospitalization during acute infection [30,31]. Conversely, COVID-19 vaccination has demonstrated a protective effect against PCC development [30,32]. The symptomatology of PCC is broad and non-specific, often involving fatigue, dyspnea, chest pain, myalgia, cognitive dysfunction, and psychiatric disturbances. Importantly, PCC symptoms may follow variable trajectories. Some individuals experience temporary remission followed by relapses of symptoms, while others report continuous or fluctuating symptoms for months or even years, leading to substantial functional impairment [5]. These clinical complexities underscore the necessity of understanding the underlying pathophysiological mechanisms, which are addressed in the following section.

## 3. Pathophysiological Mechanisms Underlying PCC

The pathophysiology of PCC is complex and multifactorial, involving persistent immune activation, systemic and neuroinflammation, oxidative stress, viral persistence, and vascular dysfunction. One of the central components is immune dysregulation following SARS-CoV-2 infection. Studies have reported prolonged activation of CD4^+^ and CD8^+^ T cells, along with a sustained elevation of proinflammatory cytokines and chemokines such as IL-2, IL-6, IFN-γ, and TNF-α [33,34,35,36,37]. A critical molecular mediator of this inflammatory process is the NOD-like receptor family pyrin domain-containing 3 (NLRP3) inflammasome, which amplifies cytokine release and contributes to a systemic inflammatory milieu [12,13]. These immune responses are further exacerbated by oxidative stress, due to the overproduction of ROS, primarily via NOX, which overwhelms cellular antioxidant defenses and disrupts mitochondrial integrity, promoting further tissue injury [14,15].

In addition to immune dysregulation, growing evidence supports the hypothesis that viral persistence drives prolonged symptomatology. SARS-CoV-2 RNA and proteins have been detected in nasopharyngeal and fecal samples for extended periods, sometimes beyond 45 days post-infection, particularly in severe cases [38,39]. These findings suggest that latent viral reservoirs may sustain chronic immune activation and disrupt tissue homeostasis [40,41,42]. Neuroinvasion is another relevant mechanism, with SARS-CoV-2 viral elements identified in brainstem and cranial nerve tissues [18,43,44], indicating the virus’s neurotropic potential. Moreover, peripheral immune responses may indirectly impact the Central Nervous System (CNS) through cytokine-mediated signaling, resulting in microglial activation, neurotoxicity, and cognitive symptoms such as brain fog, memory impairment, and depressive symptoms [45,46,47,48].

Neuroimaging studies have demonstrated structural alterations in PCC patients, including reductions in gray matter volume in the orbitofrontal cortex and parahippocampal gyrus several months after acute infection [49]. Experimental models further show that elevated levels of chemokines such as C-C motif chemokine ligands 11 (CCL11) can disrupt hippocampal neurogenesis, offering a mechanistic link to cognitive deficits [50]. Inflammasome activation and oxidative overload are also believed to trigger neurodegenerative cascades similar to those observed in Alzheimer’s disease [51].

Finally, vascular and endothelial dysfunction represent additional contributors to PCC pathogenesis. Endothelial biomarkers, including von Willebrand factor (VWF), remain elevated long after infection, consistent with sustained endothelial activation or injury [52]. Retinal imaging has revealed reduced capillary perfusion in PCC patients, providing visual evidence of systemic microvascular compromise [53]. Together, these findings indicate that PCC is the result of interconnected systemic and CNS-specific processes, including chronic inflammation, redox imbalance, viral persistence, and vascular impairment, all of which may be therapeutically targeted in ongoing efforts to alleviate long-term COVID-19 sequelae.

## 4. Neuroinflammation and Oxidative Stress in PCC

It is well established that following a CNS viral infection, proinflammatory cytokines and chemokines are released, resulting in neuroinflammation and neuronal damage [54]. It is noteworthy that several neurological manifestations of COVID-19, such as headache, encephalitis, dizziness, hallucinations, and malaise, are also observed in other respiratory viral illnesses, such as influenza. This suggests that the neuroinflammation caused by SARS-CoV-2 may be comparable to that induced by other common respiratory viruses [55]. However, several research groups have reported that SARS-CoV-2 may invade the CNS by infecting choroid plexus epithelial cells, thereby disrupting the blood–cerebrospinal fluid barrier and allowing for viral entry into the brain [56]. Neuroinflammation resulting from SARS-CoV-2 infection has been proposed as a key pathogenic mechanism contributing to neurological disorders. Evidence indicates that patients with COVID-19 exhibit a robust neuroinflammatory response, including elevated levels of IFN-α, IFN-γ, IL-6, interleukin-8 (IL-8), interleukin-10 (IL-10), interleukin-18 (IL-18), CXCL10, and nucleotide-binding oligomerization domain-containing protein 2 (NOD2), compared to healthy individuals [57,58,59]. Similarly, several studies have reported the overexpression of chemokines in COVID-19, such as C-C motif chemokine ligands 8 and 11 (CCL8 and CCL11), and C-X-C motif chemokine ligands 2, 8, 9, and 16 (CXCL2, CXCL8, CXCL9, and CXCL16) [60,61,62,63,64]. These findings suggest that systemic inflammation may contribute to neuropathogenesis in individuals infected with SARS-CoV-2 [55]. Several inflammatory pathways have been associated with SARS-CoV-2 infection, including nuclear factor kappa-light-chain-enhancer of activated B-cell (NF-κB) signaling, mitogen-activated protein kinase (MAPK) signaling, and the Janus kinase (JAK)/signal transducer and activator of transcription (STAT) pathway [65,66]. Following recovery from SARS-CoV-2 infection, some patients exhibit an increased risk of various neurological disorders, including ischemic and hemorrhagic stroke, cognitive and memory impairments, peripheral nervous system disorders, episodic disorders, extrapyramidal and movement disorders, mental health disorders, musculoskeletal conditions, sensory disturbances, and other conditions such as Guillain–Barré syndrome, encephalitis, and encephalopathy [67,68]. In line with these findings, Braga et al. conducted a case–control study involving individuals who experienced post-acute sequelae of mild-to-moderate COVID-19, which they termed “COVID-DC”. The study revealed that patients exhibited increased gliosis, particularly in the ventral striatum and dorsal putamen, along with symptoms such as motor slowing, anhedonia, and low motivation [69]. Gliosis may result from inflammation-related damage, suggesting that targeting this process could inform future therapeutic development [69,70]. Song et al. [8] propose that persistent cough in PCC may be attributed to a neuroinflammatory mechanism, whereby SARS-CoV-2 is recognized by vagal sensory neurons or sensory neuron-associated glial cells, potentially triggering a neuroinflammatory state. Consequently, SARS-CoV-2 infection stimulates sensory neurons, which, in turn, amplify the inflammatory response by releasing neuropeptides. This process facilitates the recruitment and activation of inflammatory cells, known as neurogenic inflammation [8,71]. Given the hippocampus’s susceptibility to SARS-CoV-2 infection, studies have shown that one year after the onset of post-COVID syndrome, patients exhibited hippocampal abnormalities and neuropsychological deficits linked to neuroinflammatory processes. Additionally, reductions in hippocampal subfield gray matter volume, microstructural integrity, perfusion, and functional connectivity were observed [72,73]. However, analysis of neuroinflammatory markers in cerebrospinal fluid (CSF), including microglial activation and blood–brain barrier disruption, in individuals with PCC did not reveal significant abnormalities [74]. Free radical mechanisms, oxidative stress, and molecular markers play a crucial role in the pathophysiology of PCC (Table 1).

Following SARS-CoV-2 infection, the body mounts an intensified immune response, resulting in excessive production of ROS, reactive nitrogen species (RNS), and reactive sulfur species (RSS) [75,76]. Nitrosative stress refers to the excessive or dysregulated production of RNA, primarily involving nitric oxide (NO^•^), which is synthesized by nitric oxide synthases (NOS). Under physiological conditions, NO^•^ functions as a crucial neuromodulator involved in processes such as vasodilation and synaptic plasticity. However, its uncontrolled production—particularly by the inducible isoform of NOS (iNOS) in activated immune cells—can lead to pathological effects [77,78]. The toxicity of NO^•^ stems from its ability to react with ROS, particularly superoxide anion (O_2_^•−^), forming a highly reactive and cytotoxic compound: peroxynitrite (ONOO^−^) [79,80]. Nitrosative stress plays a central role in neuroinflammation. Immune cells such as microglia and astrocytes, when activated by damage or pathogens, can express iNOS and produce large quantities of NO^•^ [81,82,83,84,85]. Sulfhydrative stress involves disturbances in sulfhydryl group homeostasis during cellular stress, emphasizing the roles reduced glutathione (GSH) and thiolated proteins as key elements of the antioxidant defense system. It reflects an imbalance that impairs the availability or function of these sulfhydryl groups, thereby compromising the cell’s capacity to manage oxidative and nitrosative stress [86,87]. Maintaining thiol homeostasis—particularly of GSH—is essential for neuronal health and for regulating neuroinflammatory processes. The key distinction between these processes lies in the dominant reactive species and their mechanisms of damage. Nitrosative stress involves excessive NO^•^ production and peroxynitrite (ONOO^−^) formation, which leads to biomolecular nitration and oxidation. In contrast, sulfhydrative stress—or thiol dysfunction—results from disruptions in sulfhydryl group availability and function, particularly of GSH, impairing the cell’s antioxidant and detoxification systems [88]. Both types of stress are closely interconnected and play significant roles in neuroinflammation. For example, nitrosative stress can induce thiol dysfunction (e.g., ONOO^−^ oxidizes GSH), while thiol depletion intensifies nitrosative stress by diminishing the cell’s capacity to neutralize RNS. Neuroinflammation is a multifaceted process in which nitrosative stress and thiol dysfunction serve as both drivers and outcomes of pathological progression [89].

Oxidative stress arises from a disruption in the equilibrium between the production of ROS and the antioxidant system’s ability to neutralize these species [90]. In PCC, this imbalance is associated with mitochondrial dysfunction, which contributes to excessive ROS production, exacerbates oxidative stress, and compromises the integrity of the BBB. This disruption facilitates the infiltration of pro-inflammatory cytokines and immune cells into the CNS, exacerbating neuroinflammation in PCC patients [91,92]. Supporting these findings, a study evaluating the relationship between oxidative status and the persistence of PCC symptoms in individuals with a history of mild COVID-19 infection demonstrated elevated levels of several oxidative stress markers, including malondialdehyde (MDA) and total hydroperoxide (TH), as well as a reduction in free sulfhydryl groups (R-SH). These results suggest that oxidative damage persists in affected individuals, indicating a potential role for oxidative stress mediators in the pathogenesis of PCC [93,94].

Lipid peroxidation, mediated by ROS, generates toxic compounds such as MDA and 4-hydroxinonenal (4-HNE), which disrupt cell membrane function and trigger inflammatory responses (Table 1) [76,90,95]. Concurrently, DNA oxidation leads to elevated levels of 8-hydroxy-deoxiguanosine (8-OHdG), a biomarker of DNA damage observed in various disease models associated with oxidative stress, including PCC [96,97,98,99].

**Table 1:** Molecular processes and markers involved in neuroinflammation in PCC.

Process Involved	Chemical Species Involved	Mechanism of Action
ROS	O_2_^•−^**,** H_2_O_2_**,** HO^•^	These species play a central role in neuroinflammation by inducing oxidative stress and sustained cellular damage to DNA, proteins, and lipids. They activate microglia and trigger the release of pro-inflammatory cytokines (e.g., IL-1β, IL-6), disrupt the BBB, and modify intracellular signaling pathways such as NF-κB, perpetuating the inflammatory cascade. This contributes to cognitive deficits and fatigue [98,100,101,102,103,104,105].
RNS	NO^•^, ONOO^−^	Induced primarily via iNOS activation, NO^•^ combines with ROS to form ONOO^−^, a highly reactive molecule that damages lipids, proteins, and nucleic acids. RNS sustain microglial activation, disrupt BBB integrity, and amplify neuroinflammation. Targeting iNOS or scavenging RNS may reduce these effects and alleviate PCC symptoms [99,100,101,106].
Lipid Peroxidation Products	MDA, 4-HNE	These molecules compromise neuronal membrane integrity, promote cytokine release, and worsen oxidative stress. They also interfere with cellular signaling and repair processes, contributing to progressive neuronal injury and exacerbating neurocognitive symptoms [96,107,108].
Protein Damage	Carbonylated proteins	Accumulated damaged or misfolded proteins initiate further inflammatory responses, impair cell signaling, and hinder tissue repair mechanisms. This perpetuates chronic inflammation and contributes to persistent symptoms such as cognitive impairment and fatigue [96,98].
DNA Damage	8-OHdG	Oxidative DNA lesions like 8-OHdG activate inflammatory and apoptotic signaling pathways. Impaired DNA repair under chronic inflammation conditions accelerates neuronal dysfunction and may underlie long-term cognitive deficits in PCC [98].
Proinflammatory Cytokines	IL-1β, IL-6, TNF-α, NLRP3 inflammasome activation	These cytokines are produced in excess in response to immune dysregulation and oxidative stress. They enhance BBB permeability, recruit immune cells into the CNS, and activate microglia, reinforcing neuroinflammation and oxidative damage. This contributes to cognitive dysfunction and mood disorders [99].
Neurotransmitters	Glutamate	Inflammatory processes disrupt neurotransmitter synthesis and signaling. Excess glutamate causes excitotoxicity and neuronal death, while reduced levels of serotonin and dopamine impair cognition, mood, and memory. This imbalance is central to “brain fog” and neuropsychiatric symptoms in PCC [98].
Cell Death	Ferroptosis	Ferroptosis is triggered by iron-mediated ROS accumulation and lipid peroxidation, especially when the antioxidant enzyme glutathione peroxidase-4 (GPx4) is deficient. Chronic inflammation worsens this process, leading to neuronal death and sustained neuroinflammation, thereby aggravating neurological and cognitive dysfunction in PCC [100].

Abbreviations: ROS: reactive oxygen species; O_2_^•−^: superoxide anion; H_2_O_2_: hydrogen peroxide; HO^•^: hydroxyl radical; RNS: reactive nitrogen species; NO^•^: nitric oxide; ONOO^−^: peroxynitrite; iNOS: inducible nitric oxide synthase; MDA: malondialdehyde; 4-HNE: 4-hydroxynonenal; DNA: deoxyribonucleic acid; 8-OHdG: 8-hydroxy-2-deoxyguanosine; IL: interleukin; TNF-α: tumor necrosis factor alpha; NLRP3: NOD-like receptor family pyrin domain containing 3; GPx4: glutathione peroxidase 4; BBB: blood–brain barrier.

Additionally, a reduction in endogenous antioxidants, such as GSH and GPx, has been reported [76,98,99,100,101]. Al-Hakeim et al. [76] found that a decrease in plasma total antioxidant capacity (TAC) suggests weakened antioxidant defenses, with significantly decreased levels of GPx and zinc, both key components of the antioxidant system. Exposure to the SARS-CoV-2 S1 protein has been shown to decrease levels of nuclear factor erythroid 2–related factor 2 (Nrf2), a key transcription factor that regulates antioxidant genes essential for counteracting oxidative stress. This reduction occurs in the paraventricular nucleus of the hypothalamus, an essential brain region involved in cardiovascular control, promoting neuroinflammation, oxidative stress, and increased sympathetic excitation in COVID-19 and PCC, where Nrf2 reduction is considered a molecular marker [102,103,104]. Nrf2 plays a key role in neuroinflammation, functioning as a crucial endogenous defense mechanism in the central nervous system. Its role focuses on neuroprotection by modulating oxidative stress and the inflammatory response. Nrf2 is a transcription factor activated in response to oxidative stress and inflammation [105]. Once activated, it translocates to the cell nucleus and binds to specific DNA sequences called Antioxidant Response Elements (AREs) [106]. By binding to AREs, Nrf2 activates the expression of a wide variety of genes that code for proteins with cytoprotective functions, including antioxidant enzymes such as heme oxygenase-1 (HO-1) and NAD(P)H quinone oxidoreductase 1 (NQO1), which help neutralize ROS and RNS, thereby reducing cellular damage, and include detoxifying enzymes and inflammation-modulating proteins that can suppress proinflammatory pathways such as NF-κB, decreasing the release of inflammatory cytokines [107].

Ferroptosis is an iron-dependent form of cell death characterized by the peroxidation and accumulation of lipids (Table 1). Additionally, COVID-19 induces iron dysregulation, which exacerbates neuroinflammation, promotes the accumulation of ROS, suppresses antioxidant defenses, and alters the renin–angiotensin system (RAS) [108]. Glutamate and glutamine are essential for neurotransmission and excitotoxicity, and also serve as key molecular markers of oxidative stress and neuroinflammation. In PCC, their levels are reduced, compromising neuroprotection against oxidative stress and impairing the modulation of metabotropic glutamate receptor (mGluR) signaling through xCT antiporter-mediated glutamate release [90,109]. Glutamate is initially converted to glutamine in astrocytes, released into the extracellular space, and subsequently taken up and reconverted into glutamate by neurons, a cycle that plays a vital role in maintaining excitatory neurotransmission [109].

## 5. Antioxidants in the Management of Neuroinflammatory Diseases

Oxidative stress is closely related to the pathogenesis of several diseases, particularly neurodegenerative disorders. In neurodegenerative diseases such as Alzheimer’s disease (AD), multiple sclerosis (MS), Parkinson’s disease (PD), and Huntington’s disease (HD), mitochondrial dysfunction is present and plays a crucial role in generating ROS and chronic inflammation, thereby exacerbating neuronal damage and leading to neuroinflammation [110,111].

Oxidative stress in these diseases, generated by ROS, increases the permeability of the BBB, allowing for the entry of neurotoxic molecules and inflammatory cells, which activate proinflammatory pathways, such as NF-κB, resulting in the release of inflammatory cytokines (e.g., TNF, IL-6, IL-1β, and IFN-γ) due to astrocyte and microglial activation, perpetuating a vicious cycle that exacerbates barrier dysfunction [112,113]. Accordingly, antioxidant-based therapeutic strategies, aimed at reducing oxidative stress, have shown promising results in these neurodegenerative disorders. For example, the use of N-acetyl-cysteine (NAC), cannabinoids, carotenoids, polyphenols, vitamins, or α-lipoic acid (ALA) demonstrated neuroprotective effects by reducing oxidative stress, apoptosis, and inflammation or showed immunomodulatory properties in traumatic brain injuries, PD, and AD in both experimental models and clinical studies [114,115,116,117,118,119].

Recent clinical studies have shown that antioxidant use improved some clinical symptoms in patients with neuroinflammatory diseases. It has been shown that natural products or plant extracts improve outcomes in neurodegenerative diseases. Ergothioneine, genistein, silymarin, matcha green tea, spirulina extract, pomegranate seed oil, and purified anthocyanins enhanced cognitive performance, reduced oxidative stress and inflammation in patients with cognitive impairment or dementia or AD [120,121,122,123,124,125,126]. Silymarin, NAC, ellagic acid, crocin, and melatonin also reduced lipoperoxidation and enhanced antioxidant capacity in patients with MS [127,128,129,130,131]. Crocin also improved motor function and lowered 8-OHdG levels in patients with PD [132]. The co-administration of probiotics and vitamin D reduced disease severity, anxiety, and gastrointestinal symptoms, as well as oxidative stress and inflammation, in patients with PD [133]. Other novel antioxidant formulations, such as Neuroaspis PLP 10™, a nutritional formula rich in omega-3 and omega-6 fatty acids with vitamins, delayed PD progression and improved functional capacity in patients with MS [134,135] and GranaGard^®^, a nanoformulation of pomegranate seed oil, improved or stabilized cognitive function in MS patients [136]. To date, new clinical trials are ongoing to evaluate the effects of antioxidants on neuroinflammatory conditions. The effects of vitamins E and C, ALA, black mulberry concentrate, and natural products such as edaravone and coenzyme Q10 (CoQ10) are being tested in mental status and antioxidant or inflammatory biomarkers in patients with AD [137,138,139,140]. Finally, the effect of NAC in motor function and behavioral symptoms is being tested in patients with HD [141] and in the atrophy and disease progression of MS [142].

Given the oxidative and inflammatory processes present in neurodegenerative diseases and during or after SARS-CoV-2 infection, antioxidants may offer therapeutic benefits for PCC by modulating neuroinflammatory mechanisms. Research indicates that oxidative stress, mitochondrial dysfunction, chronic inflammation, and immune dysregulation—along with neuropsychiatric and cognitive symptoms—are central to the pathogenesis of both neurodegenerative diseases and the long-term effects of SARS-CoV-2 infection [143,144,145,146]. The following section discusses shared pathophysiological mechanisms and provides evidence supporting the use of antioxidants to reduce neuroinflammation and alleviate nervous system symptoms in patients with PCC.

## 6. Antioxidants as a Possible Treatment in Neuroinflammation Caused by PCC

COVID-19 has been associated with oxidative stress, contributing to the formation of O_2_^∙−^ and H_2_O_2_ via NOX and SOD, respectively [61,147,148]. On the other hand, the Nrf2 pathway has been shown to be suppressed in lung biopsies from patients with COVID-19. Simultaneously, pharmacological inducers of Nrf2 have been shown to inhibit SARS-CoV-2 replication and attenuate the inflammatory response, supporting the role of oxidative stress in the pathophysiology of this condition [149]. Growing evidence suggests that elevated ROS production, oxidative stress, and hyperinflammation during SARS-CoV-2 infection may contribute to chronic inflammation, hypoxia, and ultimately endothelial dysfunction, contributing to long-term consequences commonly referred to as post-COVID-19 complications [150,151,152,153,154]. These findings have been confirmed in recent studies. Proteomic analyses have revealed that patients with post-acute sequelae of SARS-CoV-2 infection exhibited plasma proteomic alterations 3 to 9 months after infection, affecting proteins involved in inflammation, oxidative stress, and mitochondrial respiratory function. The authors concluded that mitochondrial dysfunction and immune-related pathways are altered in PCC [155]. In addition, serum levels of mitochondrial regulatory and inflammation-related proteins were shown to be significantly higher in patients with long-term pulmonary complications of PCC [156]. Other studies have shown that DNA damage repair products, MDA, 4-HNE, 8-isoprostane, and CRP levels are elevated, whereas selenium and selenoprotein P (Sepp1) levels are decreased in patients with PCC [93,100,157,158]. In the context of neuroinflammation, recent studies utilizing 3-Tesla proton magnetic resonance spectroscopy have shown that patients with PCC and persistent neuropsychiatric symptoms exhibit reduced concentrations of acetyl-containing compounds and the glutamate-glutamine complex in frontal white matter, as well as reduced myo-inositol levels in the anterior cingulate cortex-gray matter, indicating neuronal injury and glial dysfunction. The authors suggested a possible link to mitochondrial dysfunction and oxidative stress [159].

Accordingly, both natural and synthetic antioxidants have been used in PCC patients due to their anti-inflammatory properties, which may help reduce neuroinflammation and nervous system-related symptoms. Although there are a large number of antioxidant compounds, only those with the strongest supporting evidence will be discussed, specifically those evaluated in high-quality, low-bias clinical trials. A multicenter, double-blind, randomized, placebo-controlled clinical trial demonstrated that the compound PEA-LUT (Glialia^®^, palmitoylethanolamide co-ultramicronized and luteolin for 90 days) improved olfactory threshold in 130 patients recruited with PCC with persistent olfactory impairment [160]. Another randomized, controlled clinical trial also demonstrated that the same antioxidant compound (administered orally bid for eight weeks) reduced brain fog and normalizes GABA(B)-ergic activity and cortical plasticity in 39 patients who underwent transcranial magnetic stimulation and exhibited symptoms of fatigue and persistent cognitive difficulties following COVID-19 infection [161]. In a single-blind, randomized, controlled clinical trial conducted in 23 patients with persistent fatigue after of a post-acute COVID-19 infection showed that supplementation with the compound Bioarginina^®^ C (L-arginine and liposomal vitamin C for 28 days) improved walking performance, muscle strength, endothelial function, and reduced fatigue levels in these patients [162]. In a double-blind, placebo-controlled randomized clinical trial demonstrated that an oral antioxidant food supplement (tablets with zinc, echinacea, rosehip, vitamin C, propolis and royal jelly, 2 per day, for 2 months) reduced fatigue in 33 patients with PCC. Additionally, the antioxidant supplement also significantly reduced inflammatory markers such as CRP, the neutrophil-to-lymphocyte ratio, and the monocyte-to-lymphocyte ratio [163]. Finally, a double-blind, randomized, controlled clinical trial demonstrated that vitamin D supplementation (60,000 IU weekly for 8 weeks) alleviated anxiety and cognitive symptoms in 80 patients with PCC and with fatigue or neuropsychiatric symptoms [164].

Other clinical studies with a lower level of evidence (clinical trials with risk of bias, or cohort and case-control studies with a very low risk of bias and some probability of establishing a causal relationship) have shown that the use of antioxidant compounds such as CoQ10, oxaloacetate, a food supplement based on fermented papaya and noni and inhaled H_2_ can reduce neuropsychiatric symptoms such as fatigue and mental disturbances in PCC [165,166,167,168]. Another clinical study showed that the use of an antioxidant food supplement composed of vitamins B6, C, D, B12, and polyphenols decreased tiredness in PCC [169]. Other studies with a very low level of evidence (reviews or retrospective, observational, or pilot studies) have proposed the use of antioxidants based on plant extracts such as astragalus, Asian red salvia, and East Asian herbal medicines to alleviate brain fog in PCC [170,171] and the use of rosemary and black seed has been proposed as neuroprotective compounds to alleviate inflammatory symptoms in PCC [172,173,174]. Table 2 summarizes completed or terminated clinical trials in which antioxidant compounds have been used for neurological or psychiatric symptoms associated with PCC.

## 7. Regulatory Context of Antioxidant Use in PCC

The management of neuroinflammation in PCC remains a complex and evolving challenge. Although oxidative stress and neuroinflammation are recognized as key contributors to the neurological sequelae of COVID-19, the clinical use of antioxidants for this purpose is still under investigation and lacks regulatory endorsement. Currently, no antioxidant compounds have received official approval for the treatment of PCC [185,186]. Nevertheless, some agents—such as NAC—are authorized by regulatory agencies like the Food and Drug Administration (FDA), WHO, or European Medicines Agency (EMA) for other indications, including acetaminophen overdose and mucolytic therapy in respiratory diseases. Due to their known antioxidant and anti-inflammatory properties, these agents are often used off-label to alleviate symptoms associated with COVID-19 and PCC [186,187,188]. However, they have not been incorporated into formal clinical guidelines, such as those issued by the NICE, particularly regarding neuropsychiatric symptoms of PCC. Their application in practice is primarily based on emerging evidence and biological plausibility, rather than standardized protocols [185,189].

A representative example is vitamin D supplementation. Clinical trials have demonstrated its potential to reduce levels of interleukin-6 (IL-6), enhance the activity of the antioxidant enzyme GPx, and alleviate symptoms such as anxiety and depression in patients with PCC [20,185]. Nonetheless, vitamin D has not received regulatory approval for this indication, and its use remains limited to clinical trials or reflective medical practice [189].

In real-world settings, clinicians and patients have begun using antioxidants like ALA, NAC, melatonin, and vitamin D to modulate inflammation and oxidative stress in PCC. These compounds are also employed to supplement standard treatments—especially in the absence of well-defined protocols for neuropsychiatric symptoms—and to take advantage of their favorable safety profiles and ease of oral administration [190]. Although promising, these interventions are not substitutes for conventional antiviral or immunomodulatory therapies. Their use occupies a regulatory gray area: they are neither formally approved nor integrated into official clinical guidelines, but are applied out of clinical necessity—particularly in managing persistent symptoms of PCC where formal therapeutic options remain limited [186,189]. This underscores the urgent need for well-designed clinical trials to determine whether these agents warrant inclusion in standardized treatment protocols.

It is also important to consider potential safety concerns. Although antioxidants are promising, inappropriate use—particularly at high doses or in combination—may lead to adverse effects. NAC, typically administered orally at doses of 1800 to 3600 mg/day, is generally well tolerated in the short term. However, higher doses may result in nausea, vomiting, skin rash, pruritus, erythema, and, when administered intravenously, anaphylactoid reactions such as rash, bronchospasm, and hypotension, particularly in individuals with asthma or allergic predispositions [187]. Extremely high intravenous doses (e.g., 100 g) have been linked to serious complications such as hemolysis, thrombocytopenia, acute kidney injury, and death [187]. Moreover, NAC may potentiate the effects of nitrates, which can lead to hypotension in patients concurrently treated with nitroglycerin [191,192].

Similarly, vitamin D supplementation, although generally safe, carries a risk of hypercalcemia, particularly in patients with impaired renal function or those taking high doses without medical supervision. Symptoms of toxicity include nausea, polydipsia, polyuria, dehydration, confusion, and, in severe cases, acute kidney damage [193,194]. In addition, prolonged high-dose antioxidant use may disturb redox balance—a phenomenon known as “antioxidant stress”—which could impair immune responses reliant on ROS [195,196,197].

Both NAC and vitamin D may also interfere with the absorption or metabolism of other medications. NAC’s redox-sensitive and thiol-dependent pathways require careful consideration when used alongside other treatments [198], and vitamin D has been shown to reduce plasma concentrations of atorvastatin and increase the risk of hypercalcemia when co-administered with calcium or thiazide diuretics [199]. Given the lack of clinical guidelines addressing dosage, duration, and safety monitoring of antioxidants in PCC, future research must prioritize the assessment of adverse events, drug interactions, and contraindications to ensure a favorable benefit-risk profile.

## 8. Conclusions

PCC often manifests with persistent neurological and psychiatric symptoms linked to ongoing neuroinflammation, oxidative stress, mitochondrial dysfunction, and immune dysregulation. In this review, we have explored the biological foundations that underlie the potential of antioxidants as adjuvant interventions for these neuropsychiatric sequelae.

A broad spectrum of antioxidant agents, including NAC, vitamins D and C, flavonoids, polyphenols, traditional herbal remedies, and Nrf2 pathway activators, have exhibited anti-inflammatory and neuroprotective effects in both preclinical models and early clinical studies. Nevertheless, the evidence remains preliminary and somewhat fragmented. Most trials are small, of short duration, and characterize heterogeneous patient groups, which complicates efforts to establish clear links between treatment and outcomes, optimal dosage, or duration of therapy. Moreover, while some of these compounds have regulatory approval for other medical conditions, none are formally sanctioned for PCC, and their use in this context remains off-label, often grounded in mechanistic rationale rather than definitive clinical data.

Equally important are safety considerations. The use of antioxidants at high doses or over extended periods may disrupt redox balance, alter immune responses, and interact with conventional therapies used in PCC management. There are currently no standardized clinical guidelines recommending their use, underscoring the need for a cautious, patient-centered approach.

Looking ahead, the field would greatly benefit from well-designed, adequately powered randomized controlled trials that evaluate clearly defined antioxidant regimens using consistent clinical endpoints and biomarkers. These studies should not only measure efficacy and long-term safety, but also focus on patient-reported outcomes and biological mechanisms of action. To support clinical translation, a coordinated effort across disciplines will be essential to establish regulatory standards and evidence-informed guidelines.

Ultimately, antioxidant-based therapies hold genuine promise for managing neuroinflammatory complications in PCC. Nevertheless, to transition from experimental use to standard clinical practice, we need a robust evidence base that convincingly demonstrates both benefit and safety. Advancing in this direction, moving from hypothesis to validated therapeutic options, will be crucial in improving the long-term care of patients affected by the lingering effects of COVID-19.

## Figures and Tables

**Table 2:** Completed or terminated clinical trials investigating antioxidant compounds for neuropsychiatric symptoms in PCC.

Protocol Name	Study Information	Intervention	Neurological Condition PCC Associated Under Evaluation.	Results
Magnesium and Vitamin D Combination for Post-COVID Syndrome. NCT05630339 [175]	Interventional double-blinded randomized parallel assignment placebo-controlled study. Double masking. Phase Not applicable Sponsor: Coordinación de Investigación en Salud, Mexico State: Completed N = 150	Magnesium chloride: 650 mg capsule containing 340 mg magnesium chloride to be taken twice daily with food. Vitamin D: 4000 IU vitamin D tablets, one tablet administered at bedtime.	Fatigue, anxiety, depression for 4 months.	No results posted
Feasibility Study of Cannabidiol for the Treatment of Long COVID. NCT04997395 [175]	Interventional Single group assignment. Without masking Phase 2 Sponsor: Bod Australia State: Completed N = 12	The treatment consists of cannabidiol (CBD) (MediCabilis 5%) administration. The first 2 weeks at a dose of 1 mL twice daily (total dose 2 mL = 100 mg CBD and 4 mg of tetrahydrocannabinol, THC). There will be an option to further increase the dose up to a total dose of 3 mL per day (150 mg CBD and 6 mg THC) at the one-month follow-up visit.	Fatigue, pain, anxiety, depression, sleep quality for 20 weeks.	No results posted
Effects of PEA-LUT on Frontal Lobe Functions and GABAergic Transmission in Long-COVID Patients (PL-PC19). NCT05311852 [176]	Interventional double-blinded randomized parallel assignment placebo-controlled study. Double Masking. Phase not applicable Sponsor: Hospital of Vipiteno, Sterzing, Italy State: Completed N = 34	Granulated PEA-LUT dosage will be 700/70 mg 2 times/day.	GABA-ergic neurotransmission, synaptic plasticity, cognition, and fatigue for 8 weeks.	No results posted
Clinical Trial of Niagen to Examine Recovery in People With Persistent Cognitive and Physical Symptoms After COVID-19 Illness (Long-COVID). NCT04809974 [177]	Interventional double-blinded randomized parallel assignment placebo-controlled study. Quadruple masking. Phase 4 Sponsor: Clinical Translational Unit Research, USA State: Completed N = 37 (intervention) N = 21 (placebo)	Participants will take 2 g of Niagen (vitamin B3) capsules daily.	Cognitive functioning, depression and anxiety for 22 weeks.	The patients showed improvement in memory, attention, language and visuospatial functioning and an improved fatigue, depression and anxiety. Some patients possibly or definitively showed adverse effects: bruising on legs (1), nausea (5), heartburn/reflux (4), cold sweats (1) constipation (1), gastrointestinal discomfort (5) joint aching (1), finger stiffness (1), muscle cramps (1), headaches (3), dizziness and vertigo (3), needle prick sensations (1), worsening sleep (1), rash (2), acne (1), dry skin (1), skin rashes (1) and flushing/facial flushing (2). The authors acknowledged the small sample size, COVID-19 infection status was based on self-report and only inclusion of individuals experiencing brain fog as limitations of the study.
Pilot Study Into LDN and NAD^+^ for Treatment of Patients with Post-COVID-19 Syndrome. NCT04604704 [178]	Interventional single group assignment. Without masking (open label) Phase 2 Sponsor: AgelessRx State: Completed N = 36	Low Dose Naltrexone (LDN) will be used at a dosage of 4.5 mg/day, which will be taken orally as tablets. NAD^+^ will be administered using the IontoPatch iontophoresis patch containing 400 mg of nicotinamide adenine dinucleotide (NAD^+^) solution, which is worn on the skin once a week for 4–6 h.	Fatigue and quality of life for 12 weeks.	The patients showed improvement in fatigue symptoms and in physical and mental health. Patients showed skin irritation (11 cases) as an adverse effect.
Feasibility Pilot Clinical Trial of Omega-3 Supplement vs. Placebo for Post-COVID-19 Recovery Among Health Care Workers. NCT05121766 [179]	Interventional randomized parallel assignment placebo-controlled. Quadruple Masking Phase 1 Sponsor: Hackensack Meridian Health State: Terminated N = 7 (intervention) N = 10 (Placebo)	Omega-3 (Eicosapentaenoic acid, EPA + docosahexaenoic acid, DHA)—Dose is 2100 mg per day via 3 mini-capsules, 2×/day (a total of 6 mini-capsules per day). Each capsule contains 252 mg of EPA and 102 mg of DHA.	Fatigue, anosmia and ageusia for 12 weeks.	The patients did not show improvement in fatigue, anosmia and ageusia. Only one patient showed excessive fatigue as adverse effect.
Evaluation of Treatment with Viusid in Post-COVID-19 Syndrome. NCT06437210 [180]	Interventional randomized single group assignment. Quadruple masking. Phase not applicable Sponsor: Catalysis SL Status: Completed N = 200	Oral administration of Viusid, a nutritional supplement containing ascorbic acid, zinc and glycyrrhizic acid, oral solution 30 mL 3 times daily with meals (breakfast, lunch, and dinner).	Fatigue, pain, insomnia, depression, anxiety, headache, anosmia and ageusia for 30 days.	No results posted
COVID-19 Sequelae: Treatment and Monitoring. A Dietary Supplement Based on Sea Urchin Eggs With Echinochroma A. NCT05531019 [181]	Interventional blinded randomized parallel assignment placebo-controlled study. Quadruple masking Phase no applicable Sponsor: Fernando Saldarini, Hospital Donación Francisco Santojanni Status: Completed N = 54	Dietary supplementation with 0.025% Echinochrome A (ingested 3 mL twice daily) and 1.5 mg daily consumption of sea urchin egg extract (*Arbacia dufresnii*).	Headache, sleep disorders, dysgeusia, myalgia, arthralgia, sleep quality, fatigue, change in emotions, anxiety and depression for 90 days.	No results posted
Use of 1-MNA to Improve Exercise Tolerance and Fatigue in Patients After COVID-19. NCT04961476 [182]	Observational and prospective Phase not applicable Sponsor: Medical Center, Saint Family Hospital, Poland Status: Completed N = 50	1-Methylnicotinamide (1-MNA) supplementation.	Fatigue for 1 month.	No results posted
Olfactory Disfunction and Co-ultraPEALut. NCT04853836 [183]	Interventional double-blinded randomized parallel assignment placebo-controlled study. Double masking Phase 4 Sponsor: Arianna Di Stadio, University of Perugia Status: Completed N = 200	co-ultraPEALut (Glialia^®^, 700 PEA + 70 Luteolin) 1 sachet daily.	Parosmia for 90 days.	No results posted
Examining the Function of Cs-4 on Post-COVID-19 Disorders. NCT06054438 [184]	Interventional randomized crossover assignment. Without masking (open label) Phase not applicable Sponsor: Status: Completed N = 110	Participants will take one capsule (0.4 g) of the Chinese medicine nutritional supplement fermented by Cordyceps 4 times a day.	Insomnia, fatigue, anxiety and depression for 12 weeks.	No results posted
